# Clinical impact of clonal hematopoiesis on patients with solid tumors: a systematic review and meta-analysis

**DOI:** 10.3389/fonc.2026.1770012

**Published:** 2026-03-13

**Authors:** Vlad M. Croitoru, Adina Turcu-Stiolica, Adina Emilia Croitoru, Doru Paul, Razvan Iacob, Alina Daniela Tanase, Adrian Saftoiu, Irina Sandra, Cristiana Tanase, Irina M. Croitoru-Cazacu

**Affiliations:** 1 Department of Oncology, Fundeni Clinical Institute, Bucharest, Romania; 2 Faculty of Medicine, Titu Maiorescu University, Bucharest, Romania; 3 Department of Pharmacoeconomics, Faculty of Pharmacy, University of Medicine and Pharmacy of Craiova, Craiova, Romania; 4 Department of Health Economics and Outcomes Research, Faculty of Medicine, University of Medicine and Pharmacy “Iuliu Haţieganu”, Cluj-Napoca, Romania; 5 Faculty of Medicine, University of Medicine and Pharmacy Carol Davila, Bucharest, Romania; 6 Division of Hematology and Medical Oncology, Department of Medicine, Weill Cornell Medicine/New York-Presbyterian, New York, NY, United States; 7 Department of Gastroenterology, Fundeni Clinical Institute, Bucharest, Romania; 8 Bone Marrow Transplant Unit, Fundeni Clinical Institute, Bucharest, Romania; 9 Department of Gastroenterology, Elias Emergency University Hospital, Bucharest, Romania; 10 “Nicolae Cajal” Medical Institute, Titu Maiorescu University, Bucharest, Romania

**Keywords:** cardiovascular events, CHIP, clonal hematopoiesis, prognosis, solid tumors, survival

## Abstract

**Introduction:**

Clonal hematopoiesis (CH) is a common age-related phenomenon associated with an increased risk of hematologic malignancies and cardiovascular disease. Its prognostic significance in patients with solid tumors remains unclear. The aim of this meta-analysis was to evaluate the association between CH and clinical outcomes, including overall survival (OS), progression-free survival (PFS), cardiovascular events, and all-cause mortality in patients with solid tumors.

**Methods:**

We conducted a meta-analysis according to PRISMA guidelines, including 21 studies comprising 4845 patients with CH and 63557 patients without CH. Hazard ratios (HRs) and odds ratios (ORs) with 95% confidence intervals were pooled using random-effects or fixed-effects models as appropriate, based on assessments of between-study heterogeneity.

**Results:**

CH was not significantly associated with OS in patients with solid tumors (HR: 1.10, 95% CI: 0.92–1.32, *p* = 0.30). The pooled analysis using a random-effects model suggested a trend toward improved PFS in patients with CH compared with those without CH, although statistical significance was not reached (HR: 0.83, 95% CI: 0.67–1.02, *p* = 0.08). However, CH was associated with an increased mortality in patients with solid tumors, with nearly a twofold higher risk compared to those without CH (OR = 1.70, 95% CI: 1.34-2.16, *p* < 0.00001). Furthermore, CH was significantly associated with an increased risk of cardiovascular events (OR = 2.75, 95% CI: 1.38 to 5.47, *p* = 0.004).

**Conclusion:**

Our meta-analysis indicated that CH mutations have a prognostic value and are associated with a clinically meaningful increased risk of overall mortality and cardiovascular events in patients with solid tumors.

## Introduction

Clonal hematopoiesis (CH) refers to the acquisition of somatic mutations in hematopoietic stem cells that confer a growth advantage and lead to expanded mutant clones in peripheral blood (1). The risk of CH increases with age and the most commonly affected genes encode epigenetic regulators—*DNMT3A*, *TET2*, and *ASXL1 (*2). However, advanced sequencing technologies have resulted in highly sensitive detection of CH beyond these known driver genes (3). Clonal hematopoiesis of indeterminate potential (CHIP) refers to CH in individuals without cytopenias or evidence of hematologic malignancy, but who harbor mutations in genes associated with hematologic malignancies, detected at >2% variant allele frequency (VAF) (1).

Several factors, beyond age, can increase the risk of developing CHIP. Smoking has the strongest association, particularly with mutations in the *ASXL1* gene (4). Certain cancer treatments have also been linked to CHIP. Cytotoxic chemotherapy, especially platinum-based drugs like carboplatin, and topoisomerase II inhibitors, are known to increase the risk, primarily causing mutations in the *TP53*, *PPM1D*, and *CHEK2* genes (5, 6). In women with ovarian cancer, Poly-ADP ribose polymerase inhibitor therapy was also found to expand *TP53*-related CHIP clones (7, 8). Radiation exposure, even brief courses, has also been shown to increase the likelihood of developing CHIP (9, 10).

CHIP has been associated with increased risks of atherosclerosis, thrombosis, cardiovascular disease, acute kidney injury, and progression to hematologic malignancies (11–17). Preclinical studies suggests that mutant hematopoietic clones promote dysregulated inflammation, with altered cytokine signaling and hematopoietic differentiation (18).

In patients with solid tumors, CHIP appears to be more prevalent than in the general population and is frequently detected incidentally during cell-free DNA (cfDNA) testing (19). CHIP variants may also be observed at very low VAFs in tumor sequencing, likely reflecting blood contamination (20). Many CHIP-associated genes are also recurrently mutated in solid tumors, underscoring the need for careful bioinformatic filtering and variant calling (21). Without this, CHIP mutations can be misattributed as tumor-derived or germline variants when in fact the mutations are somatic alterations in leukocytes.

Recognition of CH is clinically important in patients with solid tumors for several reasons. Cytotoxic chemotherapy and radiation can selectively expand mutant clones (e.g., *PPM1D*, *TP53*) that have been linked to an increased risk for developing therapy-related myeloid neoplasms. Moreover, CH-associated inflammatory and myeloid alterations may influence treatment tolerance and survival (22). Furthermore, CH is a well-established source of false-positive results in tumor profiling and liquid biopsy assays, with direct implications for therapy selection and disease monitoring (21).

Despite rapid growth of the literature, the clinical impact of CH in patients with solid tumors varies widely. Heterogeneity in sequencing platforms and depth, gene panels, bioinformatic pipelines, and VAF thresholds, as well as differences in cancer type, prior therapies, and timing of sampling (pretreatment, on-treatment, or surveillance, contributes to inconsistent findings.

Accordingly, we conducted a systematic review and meta-analysis to quantify associations between CH and key outcomes in patients with solid tumors, including overall and progression-free survival, cardiovascular events, and risk of mortality.

## Methods

### Literature search and study selection

A systematic review and meta-analysis were conducted according to the Preferred Reporting Items for Systematic Reviews and Meta-Analyses (PRISMA) guidelines (**Supplementary Table 1**) (23). Eligible studies were identified by systematically searching electronic databases (PubMed, Scopus, and Web of Science) from inception to February 2026, using search terms related to “clonal hematopoiesis”, “cancer” or “solid tumor” and clinical outcomes of interest (e.g., “cardiovascular events,” “overall survival,” “progression-free survival”, “risk of mortality”). Additional studies were identified through manual review of reference lists. Inclusion criteria were: (1) studies reporting hazard ratios (HRs) or odds ratios (ORs) with 95% confidence intervals (CIs) comparing outcomes between individuals with and without CH; (2) adult human populations; and (3) full-text articles in English. Studies with insufficient data, or non-original data (reviews, case reports) were excluded.

### Data extraction and quality assessment

Two independent reviewers (I.M.C-C., V.M.C.) screened titles, abstracts, and full texts for eligibility, using Rayyan, and extracted data using Excel. Extracted variables included study characteristics, population demographics, number of patients with and without CH, effect estimates (HRs or ORs) and their 95% CIs, and outcomes of interest. Disagreements were resolved by consensus or a third reviewer (I.S.). The quality of included studies was assessed using Quality in Prognostic Factor Studies tool.

### Statistical analysis

The primary outcome measure was the pooled hazard ratio (HR) or odds ratio (OR) for outcome of interest in individuals with CH compared to those without. Meta-analyses were performed using RevMan (version 7.12.0) (24) and the metafor package (version 4.8-0) in R (version 4.0.0) (25) available through the Comprehensive R Archive Network (CRAN) (26). Effect estimates were extracted as reported in the original publications or calculated from raw data where available. For studies that provided HRs or ORs with 95% CIs, standard errors were derived from the CIs using established formulae. All effect estimates were log-transformed prior to pooling. Random-effects or fixed-effects models were applied as appropriate, according to the degree of between-study heterogeneity.

Statistical heterogeneity was quantified using the I² statistic, with thresholds of 25%, 50%, and 75% representing as low, moderate, and high heterogeneity, respectively. Cochran’s Q test was additionally used to assess between-study heterogeneity. Potential sources of heterogeneity were explored through subgroup and sensitivity analyses where sufficient data allowed. Studies with a Cook’s distance exceeding the median plus six times the interquartile range were considered influential. Publication bias was evaluated by visual inspection of funnel plots and, when applicable, by Rank correlation test and Egger’s test.

All statistical tests were two-sided, with p-values <0.05 considered statistically significant. Forest plots were generated to display pooled effect estimates, and funnel plots were used to assess potential publication bias.

## Results

### Search results

**Figure 1:**
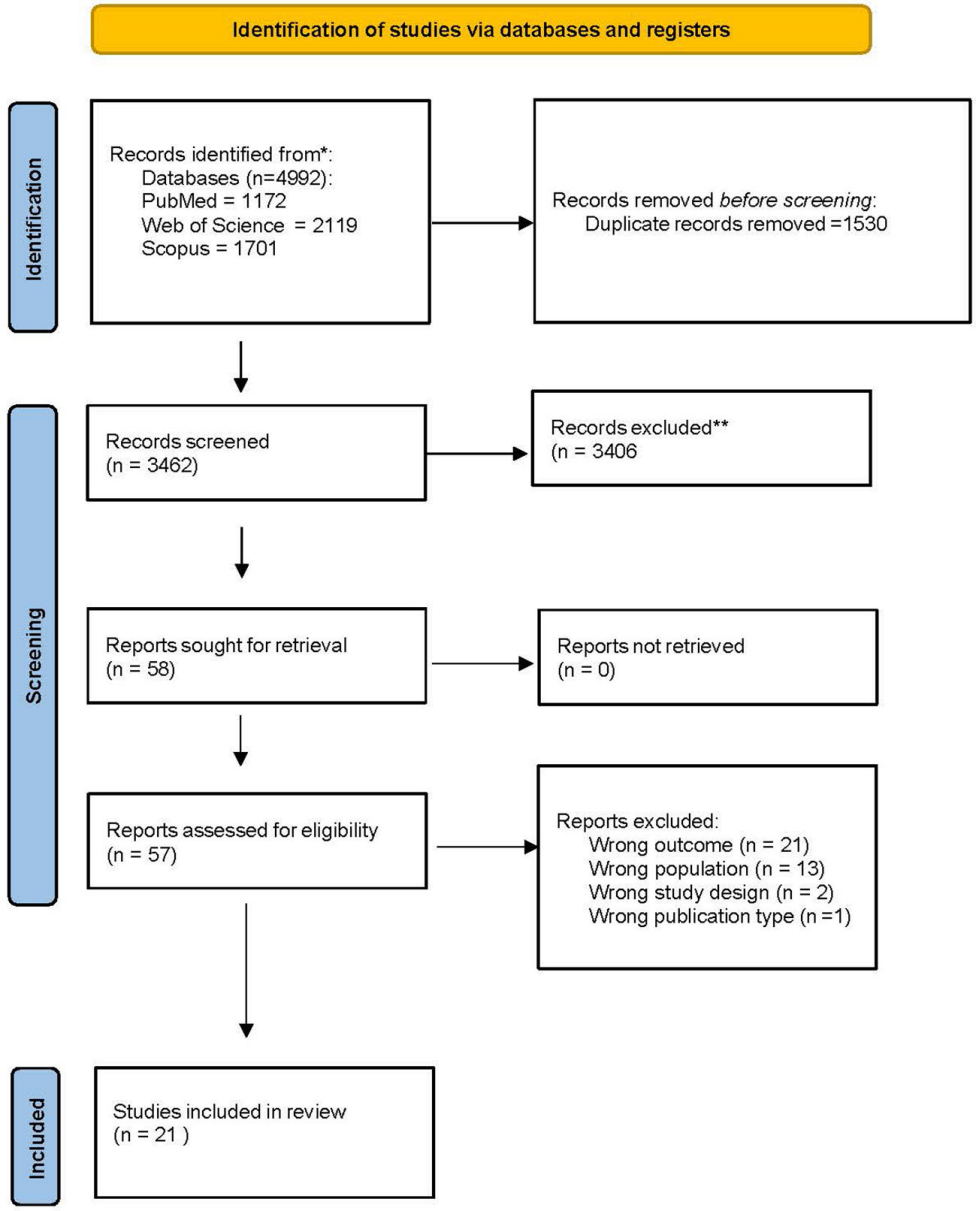
PRISMA flow diagram for the included studies that investigated clinical outcomes based on clonal hematopoiesis status in patients with solid tumors.

The study selection process, conducted in accordance with the Preferred Reporting Items for Systematic reviews and Meta-Analyses (PRISMA) 2020 guidelines, is depicted in **Figure 1** and **Supplementary Table 2**. After removing duplicate records, an initial pool of 3462 articles underwent screening based on predefined inclusion and exclusion criteria. After abstract and title screening, 58 records were selected for full-text review. Ultimately, 21 studies met all eligibility criteria and were included in the final systematic review and meta-analysis (6, 27–46).

The characteristics and reported outcomes of the included studies are detailed in a **Supplementary Table 3**. The studies comprised patient cohorts ranging in size from 39 to 49149 individuals and were published between 2018 and 2025.

### Quality of evidence

The quality assessment using the Quality in Prognostic Factor Studies (QUIPS) tool is summarized in **Supplementary Figure 1**. All studies had at least one domain rated as moderate, but only 3 of the 20 studies had a high rating for at least one domain. A moderate risk of bias was noted for in the confounding domain across all studied, which is expected as they were all observational studies that primarily managed confounding through multivariable statistical adjustment. The risk of bias in the CHIP detection domain was rated low across most studies due to the objective methods used to detect the presence of CH mutations; however, it should be noted that there is no established gold standard method for detecting CH.

### Meta-analysis of OS in individuals with and without clonal hematopoiesis

**Figure 2:**
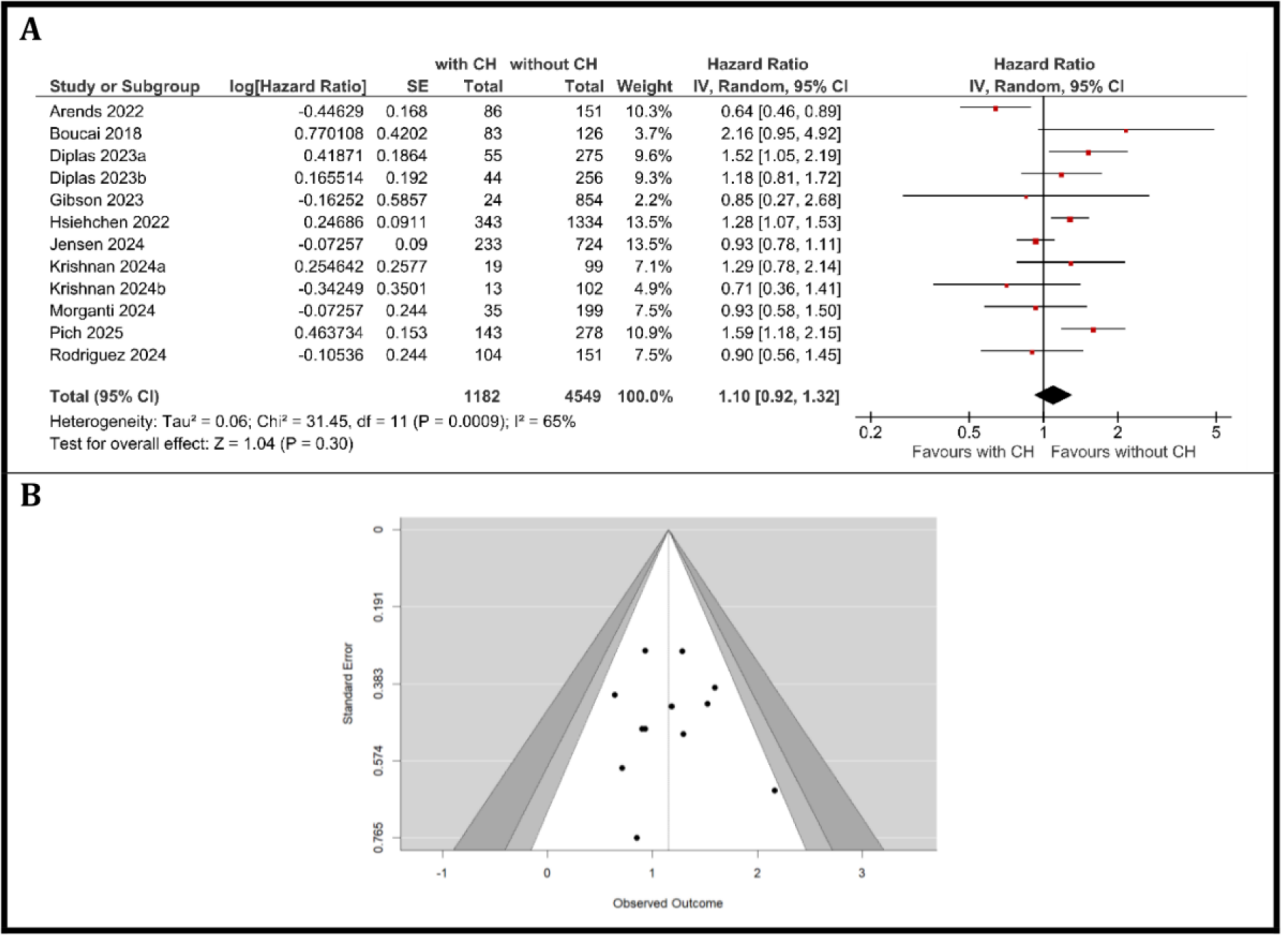
Overall survival. **(A)** Forest Plot of hazard ratios for OS comparing patients with CH versus those without CH, **(B)** Funnel Plot evaluating publication bias in studies reporting OS in patients with and without CH.

A total of ten studies with twelve cancer patients cohorts were included in the meta-analysis, comprising 1182 patients with CH and 4549 patients without CH. Between-study heterogeneity was substantial (I^2^ = 65%, τ ^2^ = 0.06, Q (11) = 31.45, *p <* 0.001). The pooled HR for OS, calculated using a random-effects model, was 1.10 (95% CI: 0.92 to 1.32, *p* = 0.30), as shown in **Figure 2A**. This suggests that CH was not significantly associated with OS in patients with solid tumors, with a non-significant trend toward increased mortality among patients with CH compared to those without.

The individual study estimates for OS varied, with HRs ranging from 0.64 (95% CI: 0.46–0.89; Arends et al. (6)) suggesting improved OS in patients with CH, to 2.16 (95% CI: 0.95–4.92; Boucai et al. (27)), indicating worse outcomes. Several studies reported wide confidence intervals that included unity, reflecting variability in study results and sample sizes. While most studies suggested a trend toward worse survival in the CH group, some confidence intervals included 1, indicating non-significant differences in individual cohorts.

Assessment of publication bias using a funnel plot (**Figure 2B**) revealed a generally symmetric distribution of studies around the pooled effect estimate, with no clear evidence of asymmetry. This suggests a low likelihood of significant publication bias in the included literature. Assessment of publication bias was performed using several complementary statistical approaches. The Rosenthal fail-safe N was calculated to be 345 (*p* < 0.001), indicating that 345 additional null studies would be required to bring the overall meta-analytic result to non-significance, suggesting the findings are robust against unpublished negative studies. Kendall’s Tau correlation (τ = 0.046, *p* = 0.837) and Egger’s regression test (intercept = 0.178, *p* = 0.859) both indicated no significant evidence of funnel plot asymmetry, further supporting the absence of substantial publication bias. Overall, these results suggest a low risk of publication bias influencing the findings of this meta-analysis.

### Meta-analysis of PFS in cancer patients with and without clonal hematopoiesis

**Figure 3:**
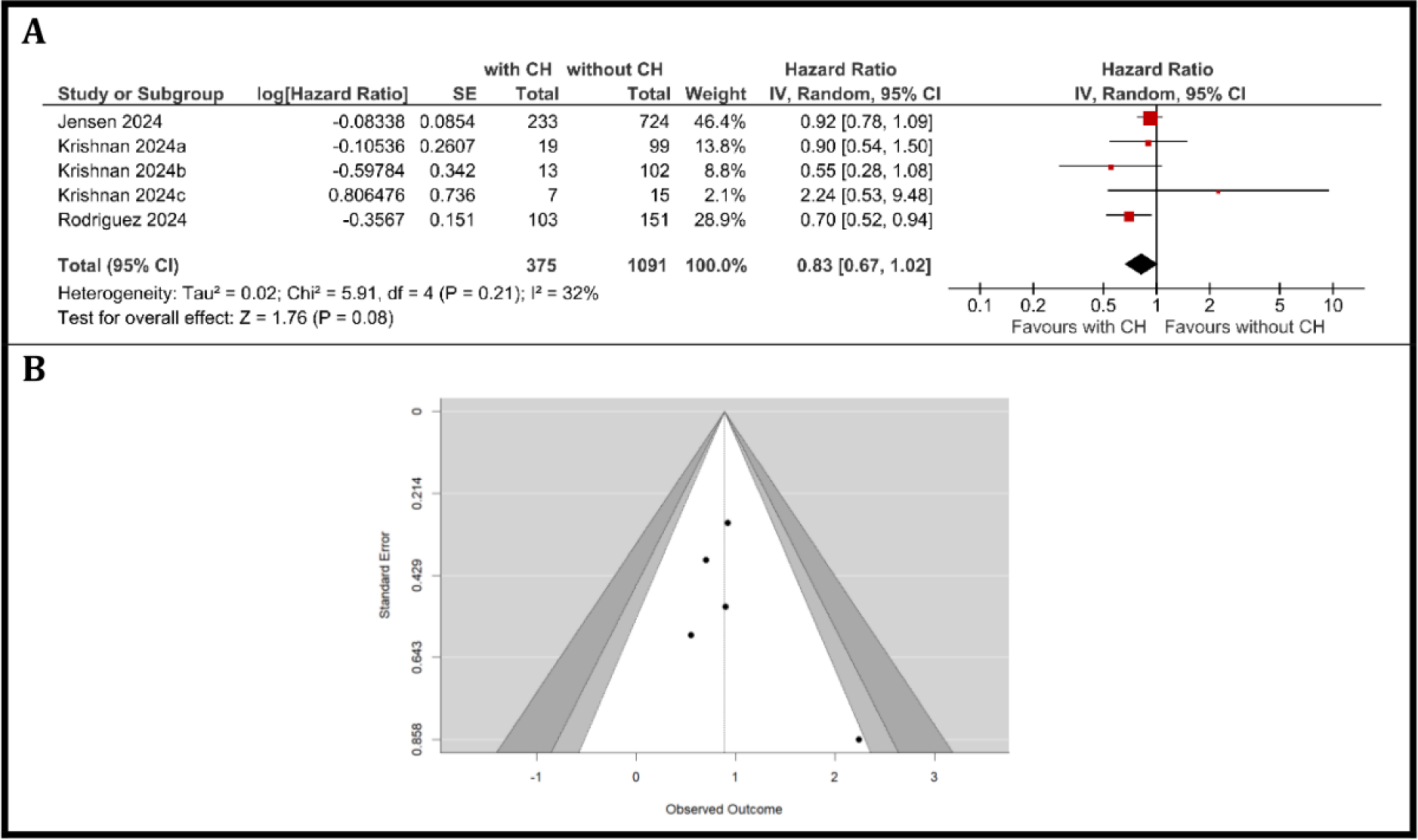
Progression-free survival. **(A)** Forest Plot of hazard ratios for PFS comparing patients with CH versus those without CH, **(B)** Funnel Plot evaluating publication bias in studies reporting PFS in patients with and without CH.

Three studies comprising five patient cohorts, including 375 patients with CH and 1091 patients without CH were included in the meta-analysis of PFS. Using the DerSimonian-Laird random-effects model, the pooled HR for PFS was 0.83 (95% CI: 0.67–1.02; p = 0.08), indicating a non-significant trend toward improved PFS in patients with CH compared with those without. Heterogeneity was moderate (I² = 32%, Chi² = 5.91, df = 4, p = 0.21; Tau² = 0.02), as shown in **Figure 3A**. Sensitivity analyses with alternative between-study variance estimators yielded consistent results. The restricted maximum likelihood (REML) estimator provided a pooled HR of 0.86 (95% CI: 0.71–1.01), while the Paule–Mandel estimator produced a pooled HR of 0.86 (95% CI: 0.54–1.17). Individual hazard ratios ranged from 0.55 (Krishnan et al, cohort b (38)) to 2.24 (Krishnan et al, cohort c (38)). The weights of the included studies ranged from 2.1% (Krishnan et al, cohort c (38)) to 46.4% (Jensen et al. (30)), reflecting variation in sample size and precision.

Among the included studies, only Rodriguez et al. (45) suggested a statistically significant association with better PFS in CH patients (HR = 0.70, 95% CI: 0.52–0.94). The overall pooled estimate suggests a non-significant difference in PFS between individuals with and without CH.

The funnel plot (**Figure 3B**) was visually inspected for publication bias. The plot demonstrated a relatively symmetric distribution of studies, with no clear evidence of publication bias; however, the small number of studies limits the interpretability of the plot. Kendall’s Tau correlation (τ = 0, *p* = 1.0) and Egger’s regression test (intercept = 0.847, *p* = 0.397) both indicated no significant evidence of funnel plot asymmetry, further supporting the absence of substantial publication bias. Overall, these results suggest a low risk of publication bias influencing the findings of this meta-analysis.

### Meta-analysis assessing the risk of cardiovascular adverse events in patients with CH compared to those without CH

**Figure 4:**
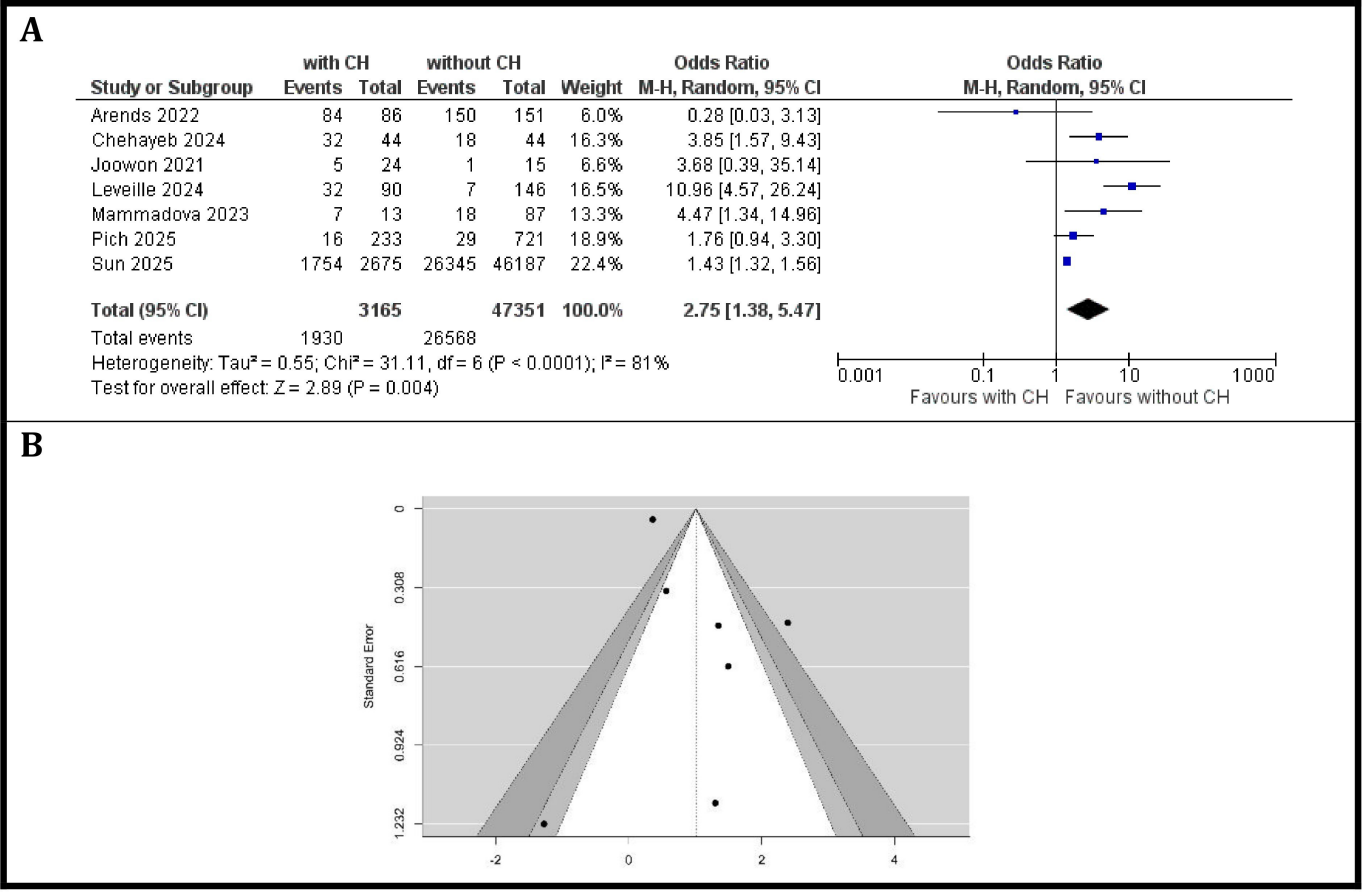
Cardiovascular events. **(A)** Forest plot of odds ratios for cardiovascular events comparing patients with CH versus those without CH, **(B)** Funnel plot evaluating publication bias in studies reporting cardiovascular events in patients with and without CH.

A total of seven studies comprising 3–165 patients with CH and 47–351 patients without CH were included in the analysis of cardiovascular events in patients with solid tumors. The incidence of cardiovascular events varied considerably across studies. The observed odds ratios ranged from 0.28 (Arends et al. (6) reported a lower risk in CH patients) to 10.96 (Leveille et al. (42) demonstrated a markedly increased risk associated with CH), with some studies showing statistically significant associations while others did not, as shown in **Figure 4A**. The pooled analysis using a random-effects model showed that the presence of CH was associated with a significant increased risk of cardiovascular events: the estimated average OR based on the random-effects model was 2.75 (95% CI: 1.38 to 5.47, *p* = 0.004). This suggests that individuals with CH have approximately a 2.75-fold higher risk of cardiovascular adverse events compared to those without CH.

According to the Q-test, the true outcomes appear to be substantial heterogeneous (Q (6) = 31.11, *p* < 0.0001, τ ² = 0.552, I² = 81%) and a random-effects model was fitted to the data. The observed heterogeneity in individual study results may be attributable to differences in study populations, or definitions of CH.

Neither the rank correlation nor the regression test indicated any funnel plot asymmetry (*p* = 0.92 and *p* = 0.81, respectively) in **Figure 4B**. Assessment of publication bias using a funnel plot (**Figure 4B**) revealed a relatively symmetric distribution of studies, although the small number of studies precludes definitive conclusions. There was no clear evidence of publication bias.

### Meta-analysis assessing the risk of mortality events in patients with CH compared to those without CH

**Figure 5:**
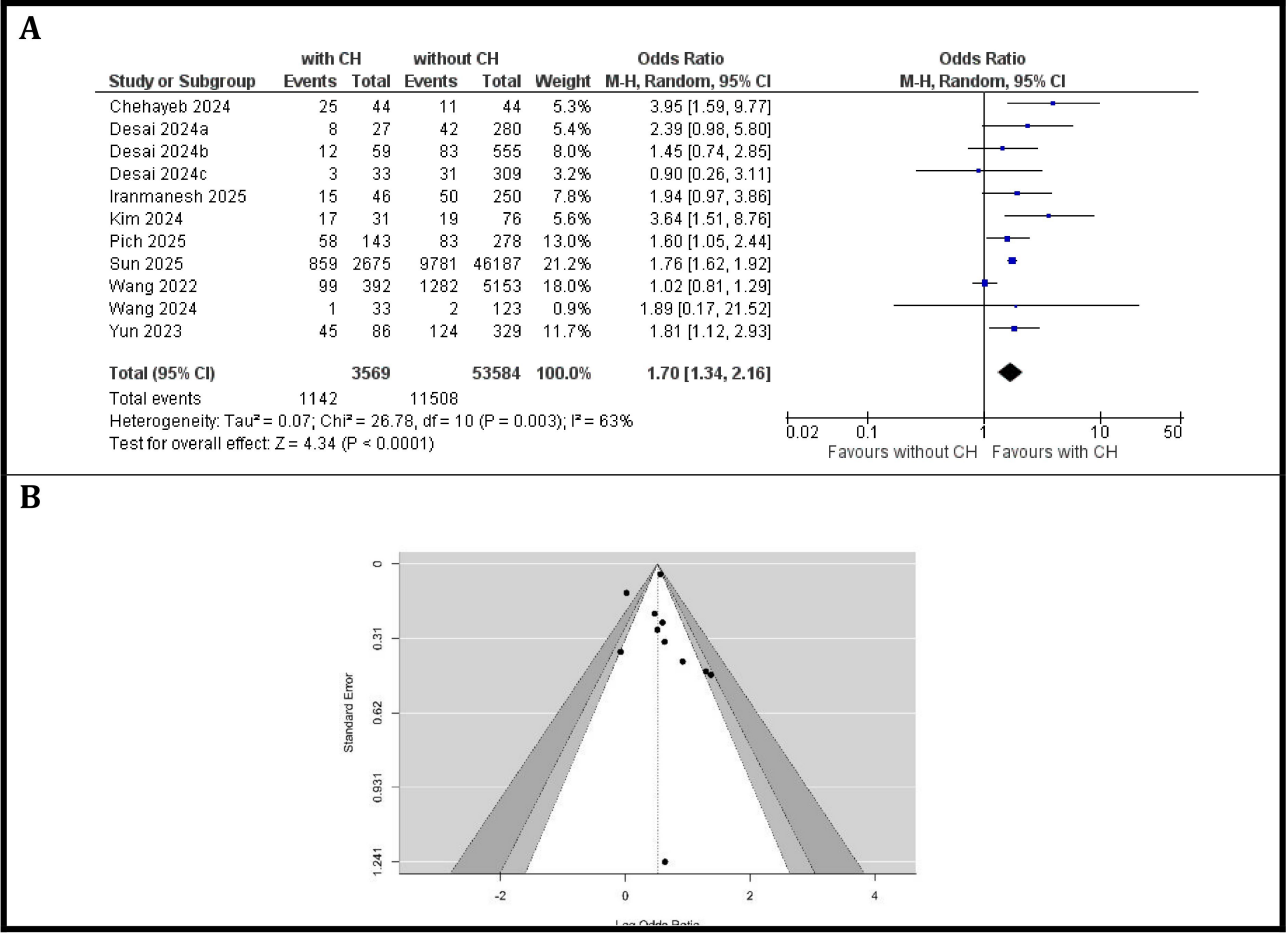
Risk of mortality. **(A)** Forest plot of odds ratios for risk of mortality comparing patients with CH versus those without CH, **(B)** Funnel Plot evaluating publication bias in studies reporting the risk of mortality in patients with and without CH.

A total of eight studies with eleven patient cohorts comprising 3–569 patients with CH and 53–584 patients without CH were included in the analysis, as presented in the **Figure 5A**. Across studies, the odds of mortality varied, with individual odds ratios (ORs) ranging from 0.90 (95% CI: 0.26–3.11; Desai et al., cohort c (39)) to 3.95 (95% CI: 1.59–9.77; Chehayeb et al. (28)).

The pooled analysis using a random-effects model demonstrated that CH was associated with a significantly increased risk of mortality in patients with solid tumors (OR = 1.70, 95% CI: 1.34–2.16; *p* < 0.0001). Between-study heterogeneity was moderate (Chi² = 26.78, df = 10, *p* = 0.003; I² = 63%). An examination of the studentized residuals revealed that none of the studies had a value larger than ± 2.78 and hence there was no indication of outliers in the context of this model. According to the Cook’s distances, two studies (Sun 2025 (46); Wang et al., 2022 (33)) could be considered to be overly influential, perhaps due to the large number of included patients (3–067 patients with CH and 51 340 patients without CH).

Visual inspection of the funnel plot from **Figure 5B** does not suggest potential asymmetry. Neither the Begg’s rank correlation test (*p* = 0.2830) nor the Egger’s regression test (*p* = 0.8234) indicated any funnel plot asymmetry.

## Discussion

The current meta-analysis comprehensively assessed the prognostic and clinical implications of clonal hematopoiesis in patients with solid tumors across 21 studies. The pooled results provide new insights into the associations between CH and key clinical outcomes including OS, PFS, cardiovascular events, and all-cause mortality. While CH was not significantly associated with OS or PFS, it was significantly linked to both an increased risk of cardiovascular events and higher odds of mortality.

The pooled hazard ratio for OS (HR = 1.10, 95% CI: 0.92–1.32) indicated no statistically significant association between CH and survival outcomes in patients with solid tumors. Although a non-significant trend toward increased mortality was observed among patients with CH, the wide confidence interval and the presence of substantial heterogeneity (I² = 65%) suggest that this relationship is more complex and potentially influenced by other factors such as tumor type, CH mutation spectrum, or treatment context. In one study (6) including patients with metastatic colorectal cancer, CH was identified as an independent predictor of better overall survival. This finding was observed even when accounting for factors like age, treatment, disease progression, and functional status. The effect was most notable in patients with DNMT3A mutations, who also showed early tumor shrinkage (6). This suggests a potential interaction between CHIP and certain cancer treatments. More research is needed to determine if CHIP can serve as a prognostic biomarker in solid tumor malignancies and how it should be considered in future clinical trials.

Interestingly, our meta-analysis showed a non-significant trend towards improved PFS in patients with CH (HR = 0.83, 95% CI: 0.67–1.02; p=0.08). The direction of effect was consistent across variance estimators (DerSimonian–Laird, REML, and Paule–Mandel), highlighting the robustness of the findings. This observation is counterintuitive and warrants further investigation, particularly to determine whether certain CH subtypes or treatment contexts might confer a selective advantage. Unlike the substantial heterogeneity observed in analyses of OS (I² = 65%), the heterogeneity for the PFS meta-analysis was moderate (I² = 32%) and could be driven by the differential impact of various cancer therapies on patients with CH. A study investigating the impact of CH on treatment outcomes found a trend toward improved PFS in CH-positive patients treated with immunotherapy (HR = 0.55, 95% CI 0.28–1.07; P = 0.079) (38). Conversely, the same study showed that CH-positive patients treated with chemotherapy had a trend toward worse PFS (HR = 1.82, 95% CI 0.98–3.38; P = 0.059) (38). The paradoxical finding of a trend toward improved PFS with CH in a pooled analysis may be explained by the complex role of CH in modulating the tumor microenvironment (TME), particularly its link to systemic inflammation. While chronic inflammation is known to contribute to tumor progression and worse outcomes in solid tumors, a specific pro-inflammatory TME may paradoxically make tumors more responsive to immunotherapies that rely on immune cell activation (47). The differential impact is further supported by research into specific CH-associated genes. For instance, mutations in *TET2*, which are common drivers of CH, have been shown to enhance the response to immune checkpoint blockade in murine models (48). This effect was linked to enhanced immune infiltration, inflammation, and T-cell activation in tumors from patients with *TET2* driver mutation-CH. Similarly, loss-of-function mutations in *DNMT3A* have emerged as a potential biomarker for increased sensitivity to PD-1/PD-L1 blockade in non-small cell lung cancer. In one study, patients with *DNMT3A*-mutated NSCLC had a higher response rate and longer PFS and overall survival when treated with immune checkpoint inhibitors (49). This suggests that specific CH mutations may be predictive of a favorable response to immunotherapy, rather than being a general prognostic indicator for survival.

An important finding of this meta-analysis is the significantly increased risk of cardiovascular events in patients with CH and solid tumors. Our pooled analysis demonstrated that CH was associated with a 2.75-fold higher risk (95% CI: 1.38 to 5.47, p=0.004) of cardiovascular adverse events. This observation aligns with a growing body of evidence highlighting the pro-atherogenic and pro-inflammatory effects of CH, particularly through driver mutations like *TET2* and *DNMT3A*, which can accelerate atherosclerotic plaque development and increase the risk of myocardial infarction and stroke (12–14, 17, 50). The wide range of reported odds ratios (ranging from 0.18 to 10.96) across individual studies underscores the complexity of this association, likely influenced by differing patient demographics, CH detection methods, and definitions of cardiovascular events. Nevertheless, the statistically significant pooled effect emphasizes the importance of screening for and managing cardiovascular risk factors in this patient population.

Furthermore, our analysis revealed a significantly increased overall mortality risk associated with CH in patients with solid tumors (OR = 1.70, 95% CI: 1.34–2.16; p<0.0001), based on eight studies. This finding, derived from a fixed-effects model, suggests that CH is an independent prognostic factor contributing to adverse outcomes. The contrast between this significant finding and the non-significant association with OS (HR = 1.10, 95% CI: 0.92-1.32, p=0.30) requires careful interpretation. The most likely explanation for the apparent discrepancy between the risk of mortality and OS meta-analyses is the different sets of studies included for each endpoint. Only one study (Pick et al.) contributed data to both analyses, while the remaining studies differed in cancer type, treatment context, follow-up duration, and reporting methodology. Thus, the differing statistical significance between overall mortality and OS should be interpreted cautiously and likely reflects methodological rather than biological factors. This discrepancy underscores the need for clear definitions of endpoints in future research.

Limitations of our meta-analysis include heterogeneity between studies. The substantial heterogeneity (I² = 32%) in OS meta-analysis was supported by the varied individual HRs for OS, with estimates suggesting improved OS (6) and estimates indicating worse outcomes (27). In the same way, the moderate heterogeneity (I² = 32%) in the PFS meta-analysis indicates some variability among included studies, potentially reflecting differences in tumor type, treatment regimen, or definitions of CH across cohorts. Notably, the study by Krishnan (cohort c) (38) reported a HR >2, favoring worse outcomes in CH patients, while other studies generally suggested HRs <1, favoring better outcomes. These discrepancies underscore the need for larger, well-characterized cohorts to delineate the clinical impact of CH more precisely. Although the overall pooled estimates trended toward benefit for CH patients, the confidence intervals crossed unity and thus preclude definitive conclusions. It remains unclear whether CH plays a prognostic or predictive role in solid tumors, and whether its impact varies depending on the tumor context or treatment exposure. We were not able to perform subgroup analyses for the reported outcomes due to limited data being available and were not able to evaluate the impact of specific CH mutations.

Overall, the findings of this meta-analysis underscore several key directions for future research. First, there is an urgent need for standardized definitions and methodologies for detecting and quantifying CH in solid tumor patients to reduce heterogeneity across studies. This includes consistent thresholds for variant allele frequency and comprehensive gene panels. Second, larger prospective studies with well-defined cohorts are necessary to validate the observed associations, particularly the intriguing trend for improved PFS and to further elucidate the interplay between CH, tumor biology, and treatment response. Finally, given the significant cardiovascular risk, clinical trials investigating tailored screening strategies and targeted interventions for cardiovascular disease prevention and management in patients with CH and solid tumors are warranted. This could lead to improved long-term outcomes for these patients. However, while CH testing may provide prognostic and cardiovascular risk information, current logistical and financial constraints may limit its immediate clinical implementation.

In conclusion, this meta-analysis indicates that CH in patients with solid tumors is significantly associated with an increased risk of cardiovascular events and overall mortality. While a non-significant trend towards improved PFS was observed, CH did not show a significant association with OS in this population. These findings highlight CH as an important comorbidity in cancer patients, particularly due to its profound impact on cardiovascular health and overall mortality. Integrating CH assessment into the clinical management of solid tumor patients may facilitate earlier identification of at-risk individuals and guide personalized therapeutic strategies aimed at mitigating cardiovascular complications and improving overall outcomes.

## Data Availability

The original contributions presented in the study are included in the article/**Supplementary Material**. Further inquiries can be directed to the corresponding author.

## Supplementary material

Supplementary Table 1 PRISMA checklist.

Supplementary Table 2 Studies excluded after eligibility assessment.

Supplementary Table 3 Characteristics of the studies included in the meta-analysis.

Supplementary Figure 1 Quality assessment. Rating for each study for each bias domain of the QUIPS tool, after consensus.
