# Supplementary material for: Clinical impact of clonal hematopoiesis on patients with solid tumors: a systematic review and meta-analysis
Source: Front Oncol. 2026 Mar 13;16:1770012. doi: 10.3389/fonc.2026.1770012 (PMC13034474; doi:10.3389/fonc.2026.1770012)
Supplement: Supplementary Table 2 — Studies excluded after eligibility assessment. [file Table2.pdf]

Supplementary Table 2. Studies excluded after eligibility assessment

Wrong outcome	Wrong population	Wrong study desig	Wrong publication type
Singh et al. Doi: https://doi.org/10.3390/ijms252011049	Hsu et al. Doi: 10.1016/j.stem.2018.10.004	Pich et al. Doi: https://doi.org/10.1038/s41467-021-24858-3	Hawking et al. doi: https://doi.org/10.1002/cam4.70792
Aldea et al. Doi: 10.1200/PO.22.00583	Boucai et al. doi: https://doi.org/10.1111/cen.14925	Maibritt Nørgaard et al. Doi: https://doi.org/10.1186/s13046-025-03356-0	
Exposito el al. Doi:10.1200/PO.23.00070	Navitski et al. doi: https://doi.org/10.1016/j.gore.2021.100873		
Hong et al. doi: https://doi.org/10.1158/0008-5472.CAN-21-1903	Musson et al. doi: https://doi.org/10.1038/s41375-023-02040-6		
Marshall et al. doi: https://doi.org/10.1002/pros.24712	Nørgaard et al. doi: https://doi.org/10.1186/s13046-025-03356-0		
Sim et al. doi: https://doi.org/10.7554/eLife.96951.3	Zhang et al. doi: https://doi.org/10.1097/JU.0000000000001878		
Slovak et al doi: https://doi.org/10.1007/s10549-009-0597-5	Cheng et al. doi: https://doi.org/10.1002/ctd2.258		

Miller et al doi: https://doi.org/10.1200/po.20.00186	Ong et al. doi: 10.1016/S2152-2650(22)01424-0		
Anandakrishnan et al. doi: https://doi.org/10.1038/s41598-023-40256-9	Olszwski et al. doi: https://doi.org/10.1111/bjh.15861		
Arends et al. doi: https://doi.org/10.1038/s41375-024-02253-3	Gillis et al. doi: 10.1016/S1470-2045(16)30627-1		
Marchetti et al. doi: 10.1097/HEP.0000000000000839	Xi et al. doi: https://doi.org/10.1016/j.tranon.2024.102242		
Magee et al. doi: https://doi.org/10.1158/1078-0432.CCR-24-3335	Tiejde et al. doi: 10.1101/2024.10.10.617685.		
Cristall et al. doi: https://doi.org/10.1158/1078-0432.CCR-24-3335	Woo et al. doi: https://doi.org/10.1158/1940-6207.CAPR-23-0342		
Kfoury et al. doi: 10.1136/ijgc-2024-005581			
Cheng et al. doi: https://doi.org/10.1002/ctd2.258			
Young et al. doi: 10.1200/PO.20.00046			
Singh et al. Doi: https://doi.org/10.3390/ijms252011049			
Aldea et al. Doi: 10.1200/PO.22.00583			

Exposito el al. Doi:10.1200/PO.23.00070			
Hong et al. doi: https://doi.org/10.1158/0008-5472.CAN-21-1903			
Marshall et al. doi: https://doi.org/10.1002/pros.24712			
