# Supplementary material for: Clinical impact of clonal hematopoiesis on patients with solid tumors: a systematic review and meta-analysis
Source: Front Oncol. 2026 Mar 13;16:1770012. doi: 10.3389/fonc.2026.1770012 (PMC13034474; doi:10.3389/fonc.2026.1770012)
Supplement: Supplementary Table 3 — Characteristics of the studies included in the meta-analysis. [file Table3.pdf]

Supplementary Table 3. Caracteristics of the study included in the meta-analysis

Study	Type of cancer	No of patients included	Thres hold VAF	Overall survival		
				No patients with CH	No patients without CH	HR (95%CI)
Pich et al 2025	Lung	421	2%	143	278	1.59, (95%CI 1.18-2.15)
Jensen et al 2024	Prostate	957	2%	233	724	0.93,(95%CI 0.78 to 1.11)
Gibson et al 2023	Breast	878	2%	24	854	0.85, (95%CI 0.27-2.69)
Hsiehchen et al 2022	Mixed	1677	2%	343	1334	1.28, (95%CI, 1.07–1.53)
Krishnan et al 2024	Colorectal	168	2%	19	99	1.29 (95%CI, 0.78–2.14)
Krishnan et al 2024	Prostate	173	2%	13	102	0.71 (95%CI, 0.36–1.42)
Diplas et al 2023	Esophageal	330	2%	55	275	1.52,(95%CI , 1.06-2.20)
Diplas et al 2023	Colorectal	300	2%	44	256	1.18 (95%CI, 0.81-1.72)
Boucai et al 2018	Thyroid	309	2%	83	126	2.16, (95%CI,0.95 to 4.94)
Morganti et al 2024	Breast	234	0.5%	35	199	0.93 (95%CI, 0.58 -1.51)
Rodriguez et al 2024	Mixed	255	1%	104	151	0.9 (95%CI, 0.5-1.3]
Arends et al 2022	Colorectal	237	1%	86	151	0.64 (95%CI, 0.46-0.89)

Study	Type of cancer	No of patients included	Thres hold VAF	Progression free survival		
				No.of patients with CH	No of patients without CH	HR (95% IC)
Jensen et al 2024	ProstatE	957	2%	233	724	0.92 (95%IC, 0.78 to 1.09)
Krishnan et al 2024	Colorectal	168	2%	19	99	0.90 (95%CI, 0.54–1.50)
Krishnan et al 2024	ProstatE	173	2%	13	102	0.55 (95% CI 0.28–1.07)

Krishnan et al 2024	Mixed	124	2%	7	15	2.24 (95%CI, 0.53–9.52)
Rodriguez et al 2024	Mixed	255	1%	103	151	0.7 [95% CI, 0.57 to 1.03]

Study	Type of cancer	Risk of mortality				
		No. of events in patients with HC	No.of patients with CH	No. of events in patients without HC	Patients without CH	HR/OR(95 %CI)
Pich et al 2025	Lung		143		278	1.59 (95%CI 1.16-2.17)
Wang et al 2022	Prostate		392		5153	1.02 (95%CI, 0.81–1.30)
Yun et al 2023	Lung	45	86	124	329	
Kim et al 2024	Breast	17	31	19	76	
Chehayeb et al 2024	Mixed	25	44	11	44	
Iranmanesh et 2025	Prostate		46		350	1.8, (95%CI, 1.1, 3.1)
Wang et al 2024	Esophageal	1	33	2	123	
Desai et al 2024	Colorectal		27		280	2.50 (95%CI 1.32–4.72)
Desai et al 2024	Breast		59		555	1.53 (95%CI 0.98–2.41)
Desai et al 2024	Mixed		33		309	0.93 (95%CI 0.56–1.52)
Sun et al 2025	Mixed	859	2675	9781	46187	1.3 (95%CI 1.22–1.40)

Study	Type of cancer	Risk of cardiovascular events				
		No. of events in patients with CH	Pacients with CH	No. of events in patients with HC	Pacients without HC	HR (95%CI)/OR (95%CI)
Pich et al 2025	Lung	16	233	29	721	1.71(95%CI, 1.20-4.5)

Mammadova et al 2023	Breast, sarcoma	7	13	18	87	4.47(95%CI, 1.34 -15.0),
Chehayeb et al 2024	Mixed	32	44	18	44	
Joowon et al 2021	Mixed	5	24	1	15	
Leveille et al 2024	Mixed	32	90	7	146	2.01(95% CI: 1.03-3.93)
Arends et al 2022	Colorectal	84	86	150	151	
Sun et al 2025	Mixed	1754	2675	26345	46187	1.07(95% CI: 1.02-1.13)
