# Supplementary figures and images for: Clinical impact of clonal hematopoiesis on patients with solid tumors: a systematic review and meta-analysis
Source: Front Oncol. 2026 Mar 13;16:1770012. doi: 10.3389/fonc.2026.1770012 (PMC13034474; doi:10.3389/fonc.2026.1770012)

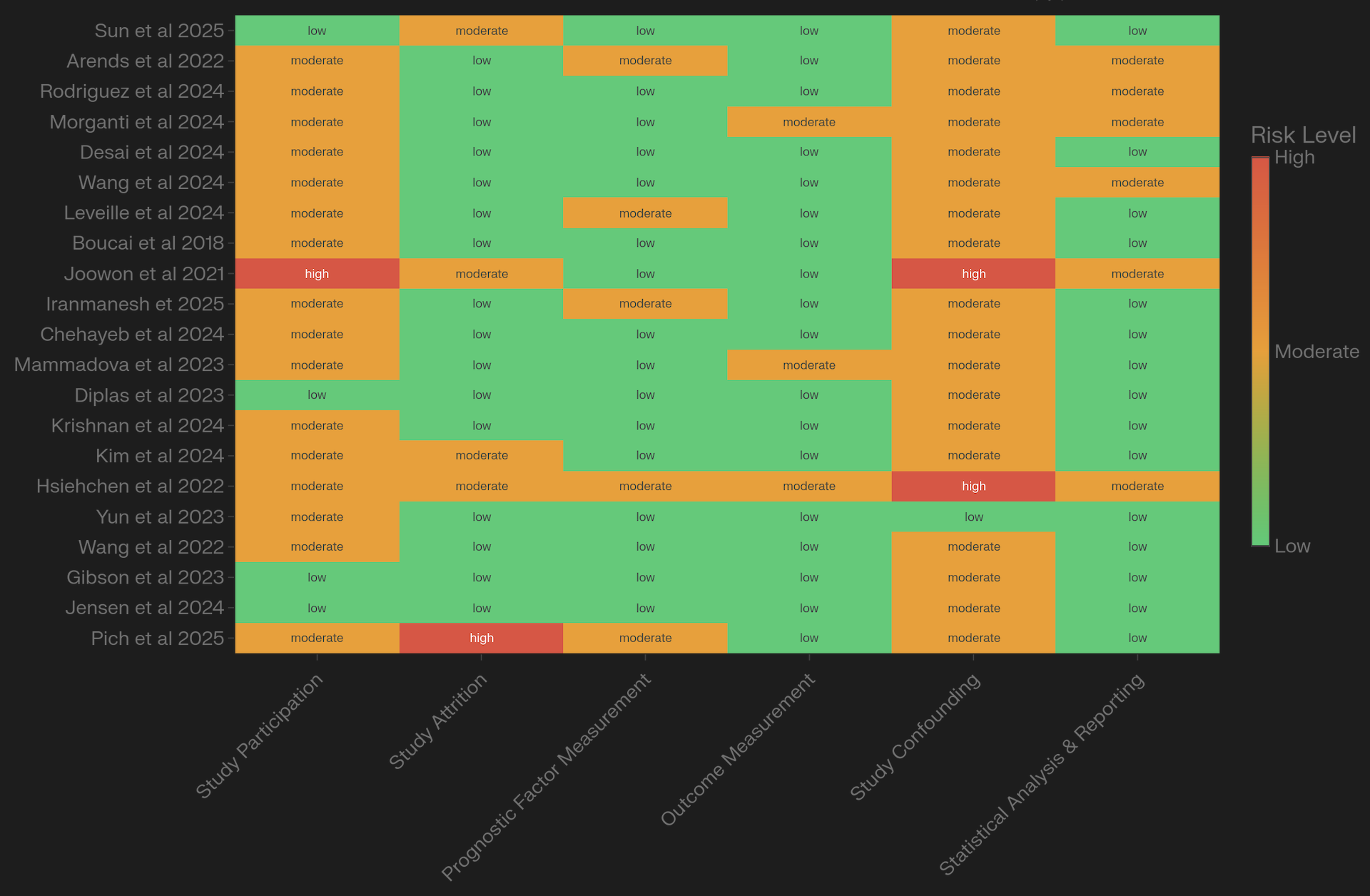

Supplement: Supplementary Figure 1 — Quality assessment. Rating for each study for each bias domain of the QUIPS tool, after consensus. [file Image1.png]
